# Deep immunophenotyping in aneurysmal subarachnoid hemorrhage: a prospective and controlled clinical study

**DOI:** 10.1186/s12974-025-03578-1

**Published:** 2025-11-29

**Authors:** Björn B. Hofmann, Dilaware Khan, Igor Fischer, Daniel Hänggi, Sajjad Muhammad

**Affiliations:** 1 Department of Neurosurgery, Medical Faculty and University Hospital Düsseldorf, Heinrich-Heine-University Düsseldorf, Düsseldorf, Germany; 2 Department of Neurosurgery, International Neuroscience Institute, Hannover, Germany; 3 Department of Neurosurgery, University of Helsinki and Helsinki University Hospital, Helsinki, Finland

**Keywords:** Aneurysmal subarachnoid hemorrhage, Angiographic cerebral vasospasm Deep immunophenotyping, Delayed cerebral ischemia (DCI), Immune response, Monocyte polarization, Neuroinflammation, T-cell subpopulations

## Abstract

**Background:**

Aneurysmal subarachnoid hemorrhage (aSAH) remains a devastating cerebrovascular condition with significant morbidity and mortality. While inflammation plays a critical role in post-hemorrhagic complications, the specific polarization dynamics of monocyte and T cell subpopulations in this context remain poorly understood. This study aims to investigate these immune cell shifts and their correlation with major complications such as angiographic cerebral vasospasm and delayed cerebral ischemia (DCI).

**Methods:**

We conducted a prospective, controlled observational single-center cohort study in a neurovascular university center of maximum care in Germany. A total of 75 patients with aSAH and 20 healthy controls were included. Blood samples were collected on days 1, 4, 7, and 11 post-bleeding. Flow cytometry was used to analyze monocyte (M1, intermediate, M2) and T cell (Th1, Th2, Th17, Treg) polarization patterns. Primary outcomes included the incidence of angiographic cerebral vasospasm, clinical DCI and/or cerebral infarction due to DCI, and clinical outcome assessed at 6 months via the modified Rankin Scale (mRS).

**Results:**

Compared to healthy controls, patients with aSAH exhibited a significant decrease in anti-inflammatory alternatively activated monocytes (6.3% vs. 3.0%; *p* = 0.04) within 24 h post-bleeding, an increase in pro-inflammatory Th17 T cells (36.3% vs. 6.2%; *p* < 0.001) and decrease in anti-inflammatory Th2 cells (45.5% vs. 75.2%; *p* < 0.001). A loss of anti-inflammatory alternatively activated monocytes was observed prior to the onset of angiographic cerebral vasospasm (3.8% vs. 2.7%; *p* = 0.031) and DCI (4.2% vs. 2.9%; *p* = 0.006). No significant correlation was found between immune cell subpopulations and long-term clinical outcomes.

**Conclusions:**

This study demonstrates a shift toward pro-inflammatory immune cell subpopulations following aSAH, with significant losses of alternatively activated monocytes preceding major complications such as vasospasm and DCI. These findings suggest that immune subpopulation profiling may hold potential as a diagnostic and therapeutic tool in the context of aSAH-related complications. Future studies should aim to clarify whether the observed peripheral immune changes reflect analogous intracerebral immune mechanisms and whether modulating these pathways can improve clinical outcomes.

**Supplementary Information:**

The online version contains supplementary material available at 10.1186/s12974-025-03578-1.

## Introduction

Aneurysmal subarachnoid hemorrhage (aSAH), accounting for 5% of strokes and predominantly affecting younger patients, remains one of the most severe cerebrovascular diseases, with high morbidity and mortality [1]. The pathophysiology of aSAH is complex [2, 3], involving the phase of early brain injury (EBI) with increased intracranial pressure, brain edema, and impaired perfusion [2–5]. This is followed by the phase of delayed cerebral ischemia (DCI), which involves angiographic cerebral vasospasm, microvascular dysfunction, and microthrombosis, resulting in a more heterogeneous and regional impairment of the brain tissue [2–5]. Despite the seemingly divergent pathophysiological processes, a prominent and consistent factor is becoming increasingly evident in both the early and late phases following aSAH: a sterile inflammation.

Although many studies have focused on detecting inflammatory mediators like cytokines in aSAH, the actual drivers of inflammation appear to be parenchymal brain cells and peripheral immune cells [6–8]. Activated resident microglia, peripheral monocytes and T cells are key in initiating and sustaining the inflammatory response [7, 8]. Studies have shown that markers such as the systemic inflammation response index, CRP levels, C-reactive protein-to-lymphocyte ratio, and the lymphocyte-to-monocyte ratio correlate with outcomes after aSAH [8–13]. Additionally, the lymphocyte-to-monocyte ratio was linked to severe complications like angiographic cerebral vasospasm [9]. As a result, it is increasingly evident that monocytes and T cells, serving as pivotal drivers of inflammation, play a critical role in the inflammatory processes following aSAH.

However, simply analyzing the overall immune cell population is insufficient. Different subpopulations of monocytes (classically activated (M1), intermediate, alternatively activated (M2)) and T cells (Th1, Th2, Th17, Tregs) have distinct pro- or anti-inflammatory effects [14–17], emphasizing the necessity for a detailed examination to comprehensively grasp the inflammatory cascades in aSAH. However, the necessary detailed deep immunophenotyping in a prospective controlled trial, most likely due to the high costs and the high effort required, has not yet been done.

The impact of peripheral monocyte and T cell subpopulations in aSAH, their temporal changes, and their correlation with complications or outcomes remain unclear. Investigating these aspects could offer insights into new therapeutic strategies for reducing aSAH morbidity and mortality. This study aims to explore the polarization of monocytes (classically activated, intermediate, alternatively activated) and T cells (Th1, Th2, Th17, Tregs) in aSAH and their correlation with outcomes and complications like angiographic cerebral vasospasm or DCI, potentially revealing new therapeutic targets.

## Materials and methods

### Ethical statement

All procedures involving human participants adhered to institutional ethical standards and the 1964 Helsinki Declaration and amendments. The study was approved by the local ethics committee (Medical Faculty, Heinrich-Heine University, Düsseldorf; study ID: 2020 − 1037_1). Written consent was obtained from capable patients immediately, or from authorized representatives for incapacitated patients. Consent from healthy controls was also obtained. The manuscript follows STROBE guidelines.

### Inclusion criteria

The study prospectively included all patients with SAH admitted to the neurovascular center from 10/2020 to 07/2022 with (1) SAH confirmed by initial non-enhanced CT, and (2) aneurysm verification by CT angiography and/or digital subtraction angiography. Exclusion criteria: (1) the patient had a known or had a history of infectious disease (to protect laboratory personnel), (2) the patient was admitted more than 24 h after the bleeding event, or (3) the patient or their relatives refused participation in the study.

### aSAH management

Patients were managed according to in-house treatment guidelines, previously described in detail [18–22].

### Study design and sample collection

Blood samples were collected within 24 h of admission (day 1) and on days 4, 7, and 11. Peripheral arterial, or in rare cases venous, blood samples were taken, totaling 10 ml of EDTA blood each time. The further processing of the samples was carried out within 2 h after sample collection.

### Survey of parameters and definitions

Patient characteristics, imaging results, medication, and medical data were recorded on a daily basis.

### Definition of primary outcomes

Clinical outcome was assessed at 6 months after hemorrhage using the modified Rankin Scale (mRS) via structured clinical follow-up. The mRS scores were stratified into favorable (0–2), unfavorable (3–5), and death (6).

DCI was defined in accordance with Vergouwen et al. [23] and categorized as either clinical DCI or cerebral Infarction due to DCI.

Clinical DCI was defined as a new focal neurological deficit or a ≥ 2-point drop in GCS lasting >1 h, not immediately following aneurysm treatment, and not explained by other causes after appropriate workup [23].

DCI-related Infarction was defined as a new cerebral infarct on CT or MRI within 6 weeks, not present on early post-treatment imaging, and not attributable to surgical or endovascular intervention [23].

Both subtypes were analyzed individually and as a combined DCI endpoint.

Angiographic cerebral vasospasm was defined as a ≥ 50% vessel narrowing on digital subtraction angiography, either relative to baseline or the contralateral side. No grading of vasospasm severity was performed.

### Sample Preparation and FACS analysis

**Fig. 1:**
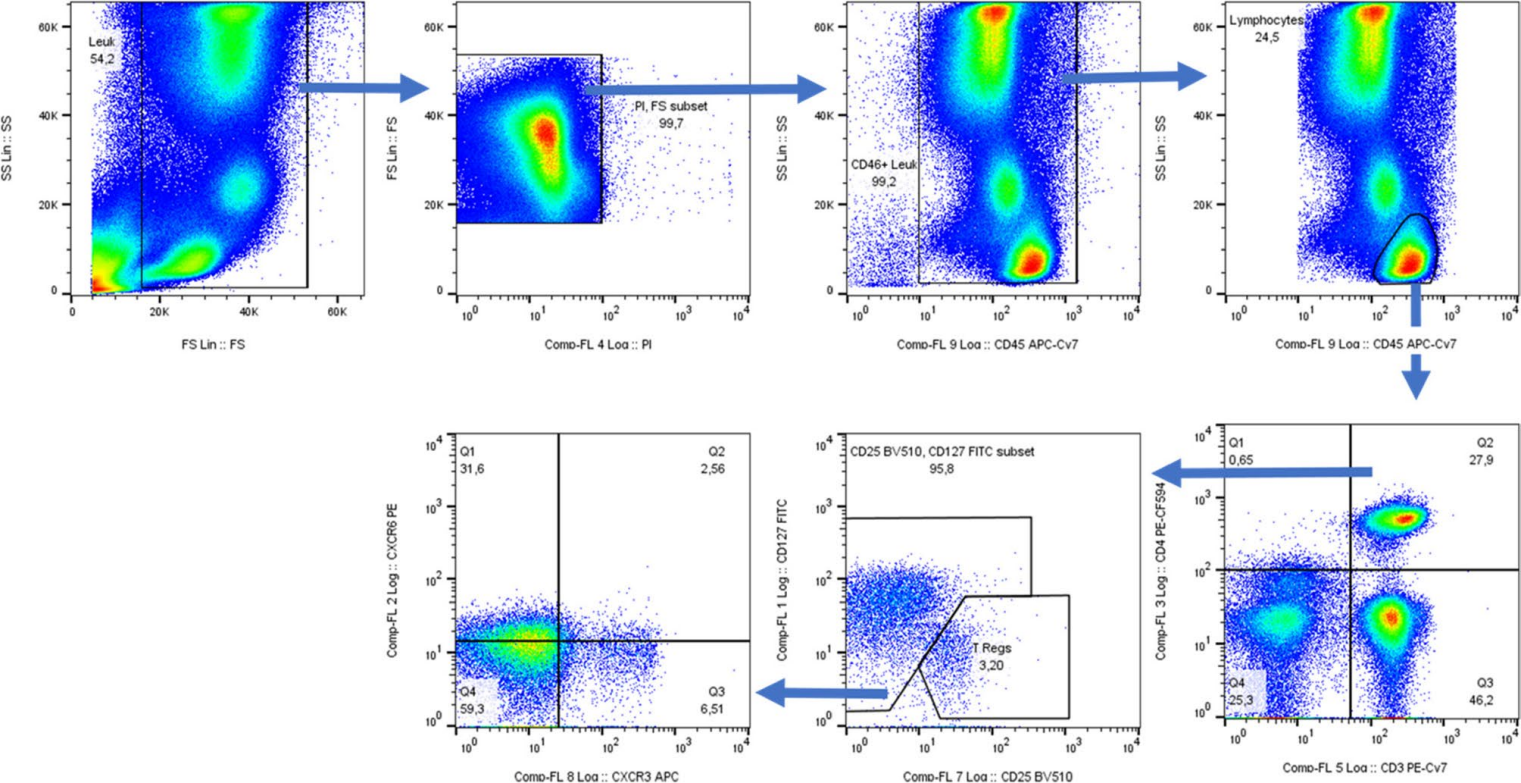
FACS gating strategy (T cells) The initial gate was made on the events with consistent flow by bivariate plot of Forward Scatter (FS) and Side Scatter (SS). Then, these events were shown as FS and SS and the gate was used to exclude erythrocytes, platelets and debris. Next the dead cells were excluded based on FS and Propidium Iodide (PI). Then, a bivariate plot of SS vs. CD45 was used to select the CD45 + leucocytes. Further, these events were displayed once again on a bivariate plot of SS vs. CD45 and then, Lymphocytes were selected from these CD45 + events by their low side scatter and high CD45 expression. These lymphocytes were then displayed as CD3 vs. CD4 plot and a quadrant gate was applied. Then, from this quadrant gate CD3 + CD4 + events were selected as CD4 + T cells. CD4 + T cells were then displayed on a CD25 vs. CD127 plot and CD25hiCD127lo events were gated as Tregs. Then, the events other than Tregs were selected and were named as Th cells. These Th cells were further displayed on a bivariate plot of CXCR3 vs. CCR6. Then, a quadrant gate was used to identify CXCR3 + CCR6- cells as Th1, CXCR3- CCR6- cells as Th2 and CXCR3- CCR6 + Th17 cells

Blood samples from healthy volunteer patients without aneurysms and without infection in the last 2 months were used as controls. To minimize batch effects, all blood samples, both from patients and healthy controls, were drawn in a narrow morning time window and processed using the same standardized protocol by a single trained operator. Briefly, the peripheral EDTA anticoagulated blood (3 ml) was lysed with erythrocyte lysis buffer (Cat# 00–4300−54, Thermo Fisher) at room temperature. After erythrocyte lysis, cells were centrifuged at 350 g for 5 min at 4° C and washed with 2 mL of an ice-cold 10% FBS (Cat# F7524, Sigma Aldrich) in PBS (Cat# 14040091, Thermo Fisher). After washing the cells, cells were resuspended in 1 mL of an ice-cold 10% FBS in PBS. The cells were counted using Trypan blue (Cat# 15250061, Thermo Fisher) and Neubauer chamber and adjusted to a final concentration of 2 million cells per 100 µL ice-cold 10% FBS in PBS. Then, 100 µL aliquots of the cells were dispensed into 5 ml flow cytometry tubes (Lot# 11021083, Corning Science). The panel of anti-human antibodies consisted of CD45, CD14, and CD16 for monocytes and CD45, CD4, CD3, CD127, CD25, CD183, CD186 (CXCR6) for T-cells (antibody details are depicted in Supplementary Tables 1 and 2). Antibody staining of the cells was performed at 4 ° C on ice. After incubation, cells were washed with 2 mL ice-cold 10% FBS in PBS and resuspended in approximately 500 µL of ice-cold 10% FBS in PBS. To discriminate between live and dead cells, 5 µL of Propidium Iodide Solution (Cat# BLD-421301, BioLegend (Via Biozol)) was added to each sample and incubated for 4 min on ice. After that, the samples were washed twice with ice-cold 10% FBS in PBS. The cells were resuspended in 100 µL of an ice-cold 10% FBS in PBS. To fix the cells, 5 µL of 16% PFA was added to each sample. The FACS analysis was performed as early as possible. For both panels, more than 500 thousand events were acquired for stained cells. The data was acquired on CyAn ADP High-Performance Flow Cytometer (Beckman Coulter) using the software Summit 4.3. For details of the gating strategy of the T cell subtypes, see Fig. 1. Monocytes were classified into classical monocytes (CD14 + + CD16-), intermediate monocytes (CD14 + + CD16++) and non-classical monocytes (CD14-CD16++) [24]. Unstained, single-stained, and FMO were used for performing the compensation. Unstained samples were used to assess autofluorescence. Single-stained controls for each fluorochrome were used to construct the compensation matrix and correct for spectral overlap. FMO controls were used for accurate gating. All controls were run under identical instrument settings as the experimental samples. The data was analyzed using FlowJo software version 10.8.1.

### Statistical analysis

Clinical data, obtained from the electronic medical records system, were merged with the laboratory data based on the patients’ ID. Laboratory values between different groups were compared using ANOVA if the number of groups was three or more, and using independent samples t-test for two groups. If ANOVA showed significant differences between groups, post-hoc t-test was used to identify the differing group(s). Patient outcome was quantified using modified Rankin scale at discharge and 6 months after bleeding, reduced to three levels: 0–2 = favorable outcome, 3–5 = poor outcome, 6 = death. As the low number of time points and their unequal spacing didn’t allow for time series analysis, the development of laboratory values over time was analyzed using ordinary least squares linear regression. To better model intra-individual trajectories, we additionally employed linear mixed-effects models with patient ID as a random intercept. To adjust for potential confounders in the association between immune profiles and clinical complications, we performed multiple logistic regression analyses with the presence of angiographic cerebral vasospasm, clinical DCI, cerebral infarction due to DCI, or overall DCI as dependent variables. Models included alternatively activated monocytes as the primary independent variable and were adjusted for age, sex, WFNS grade at admission, and modified Fisher grade. Missing data were removed as required for each analysis. To complement p-values and improve interpretability, Cohen’s d effect sizes were calculated for selected group comparisons. All calculations were performed using Python 3.9.7 and the numpy, scipy, and statsmodels packages. The significance level for this study was set to *p* ≤ 0.05.

### Statistical considerations for multiple testing

Due to the exploratory nature of the study, the small sample size, and the high dimensionality and intercorrelation of the immune parameters we decided against applying statistical corrections for multiple testing, like Bonferroni or FDR. Applying such corrections would carry a high risk of type II error and potentially obscure biologically meaningful trends, particularly in a prospective, hypothesis-generating study such as ours. Instead, we focused on consistent temporal patterns and biologically plausible associations that were supported across multiple time points and analyses.

## Results

During the study period, a total of 129 patients with aSAH were treated at our neurovascular center. Out of these, 54 either did not meet the strict inclusion criteria (first sampling within 24 h of aSAH) or the patients or their relatives declined to participate in the study. Ultimately, 75 patients were included in the analysis, and an additional 20 healthy controls were recruited and enrolled as a comparison group. The patient characteristics are detailed in Table 1. No significant associations with immune cell subtypes were observed in relation to infections requiring anti-infective or immunomodulatory treatments.

**Table 1:** Patients characteristics

	Overall (*N* = 75)
Age	
Mean (SD)	54 (± 12)
Sex	
Female	49 (65%)
Male	26 (35%)
Initial WFNS	
I	22 (29%)
II	11 (15%)
III	6 (8%)
IV	16 (21%)
V	20 (27%)
mFisher	
1	2 (3%)
2	5 (7%)
3	30 (40%)
4	38 (51%)
Aneurysm Location	
ACOM	31 (41%)
ACA	1 (1%)
PcA	3 (4%)
MCA	13 (17%)
ICA	7 (9%)
PCOM	7 (9%)
PCA	1 (1%)
BA	5 (7%)
PICA	2 (3%)
AICA	1 (1%)
Other	4 (5%)
LOS	
Mean (SD)	20.2 (± 8.4)
mRS Discharge	
0–2	38 (51%)
3–5	29 (39%)
6	8 (11%)
mRS 6 Months	
0–2	47 (62%)
3–5	12 (16%)
6	10 (13%)
Missing	6 (8%)
Angiographic Cerebral Vasospasm	
Not Present	58 (77%)
Present	17 (23%)
Clinical DCI	
Not Present	51 (68%)
Present	24 (32%)
DCI-related Infarction	
Not Present	43 (57%)
Present	32 (43%)
DCI	
Not Present	34 (45%)
Present	41 (55%)

*ACA* anterior cerebral artery, *ACOM* anterior communicating artery, *AICA* anterior inferior cerebellar artery, *BA* basilary artery, *ICA* internal carotid artery, *MCA* middle cerebral artery, *mRS* modified Rankin scale, *PcA* pericallosal artery, *PCA* posterior cerebral artery, *PCOM* posterior communicating artery, *PICA* posterior inferior cerebellar artery, *SCA* superior cerebellar artery, *SD* standard deviation, *WFNS* World Federation of Neurosurgical Societies

### Alterations in monocyte and T cell subpopulations: aSAH vs. Healthy controls

**Fig. 2:**
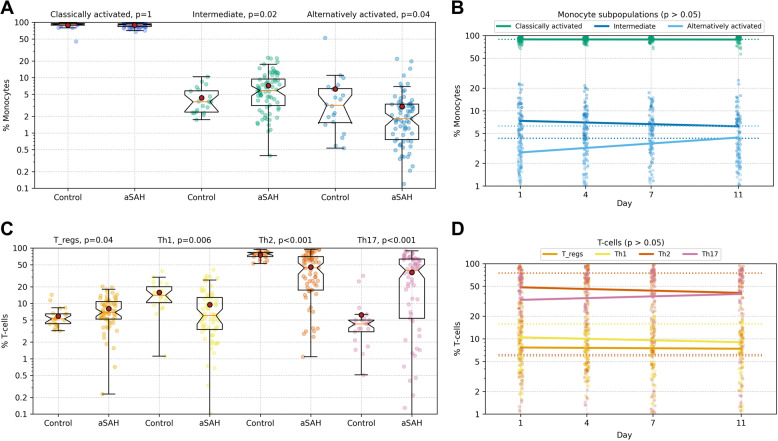
Monocyte and T cell subtypes – comparison with healthy controls and temporal dynamics in aSAH **A** Relative number of monocyte subtypes and (**C**) T cell subtypes in peripheral blood of healthy individuals (no aneurysm; no aSAH) compared to patients 24 h post-aneurysmal subarachnoid hemorrhage (aSAH), depicted as bar graphs. **B** Representation of relative number of monocyte subtypes and (**D**) T cell subtypes over time following aSAH, with measurements taken on days 1, 4, 7, and 11

In comparison to healthy controls, individuals who experienced aSAH showed a significant increase in the relative number of intermediate monocytes (4.3% vs. 7.2%; *p* = 0.02), as well as a significant relative reduction in alternatively activated monocytes (6.3% vs. 3.0%; *p* = 0.04) (Fig. 2A).

Examining T cell subtypes revealed consistent significant differences between healthy controls and those with aSAH. The relative number of T regulatory cells (5.9% vs. 8.0%; *p* = 0.04) and Th17 T cells (6.2% vs. 36.3%; *p* < 0.001) was significantly increased in patients with aSAH, while the relative number of Th1 (15.1% vs. 9.5%; *p* = 0.006) and Th2 T cells (75.2% vs. 45.5%; *p* < 0.001) showed a significant decrease (Fig. 2C). The exact numerical values are provided in Table 2.

**Table 2:** Relative distribution of monocyte subtypes

	Classically Activated Monocytes	Intermediate Monocytes	Alternatively Activated Monocytes	
	% of monocytes (± SD)			n
**Control**	89.0 (11.6)	4.3 (2.4)	6.3 (11.2)	20
**Time Course**				
Day 1	88.9 (7.1)	7.2 (5.3)	3.0 (3.9)	72
Day 4	89.1 (6.0)	7.3 (4.7)	2.9 (3.0)	74
Day 7	89.0 (6.5)	6.4 (4.1)	3.9 (3.7)	64
Day 11	88.6 (8.7)	6.2 (4.3)	4.4 (5.2)	60
**Outcome**				
Favorable	88.9 (8.1)	7.1 (5.3)	3.1 (4.6)	35
Poor	90.3 (4.6)	6.6 (4.4)	2.3 (1.4)	28
Death	83.6 (7.5)	10.2 (6.9)	5.0 (5.6)	8
**Angiographic Cerebral Vasospasm**				
Not Present	89.0 (6.0)	6.5 (3.8)	3.8 (3.4)	58
Present	88.3 (5.6)	8.2 (3.2)	2.7 (2.3)	17
**Clinical DCI**				
Not Present	88.7 (6.1)	6.6 (3.9)	4.0 (3.5)	51
Present	89.1 (5.5)	7.6 (3.4)	2.5 (2.0)	24
**DCI-related Infarction**				
Not Present	89.0 (5.9)	6.2 (3.3)	4.1 (3.5)	43
Present	88.6 (5.9)	7.8 (4.2)	2.8 (2.5)	32
**DCI**				
Not Present	89.6 (5.4)	5.6 (2.7)	4.3 (3.7)	34
Present	88.2 (6.2)	8.0 (4.2)	2.9 (2.6)	41

Representation of the relative proportions of monocyte subgroups among all monocytes in percentage ± *SD* standard deviation. Outcome = mRS 6 months post bleeding (favorable = 0–2; poor = 3–5, death = 6). *DCI* delayed cerebral ischemia, *n* number of patients

### Temporal dynamics in monocyte and T cell subpopulations in aSAH patients

Over time following aSAH, a non-significant trend was observed for monocytes, indicating a relative decrease in intermediate monocytes (day 1 vs. day 11; 7.2% vs. 6.2%; *p* = 0.115) and a relative increase in alternatively activated monocytes (day 1 vs. day 11; 3.0% vs. 4.4%; *p* = 0.136).

To better account for intra-individual variability and irregular spacing of timepoints, we additionally performed linear mixed-effects modeling. This confirmed a significant increase in alternatively activated monocytes (β₁ = +0.14, 95% CI [0.05–0.23], *p* = 0.003) and a significant decrease in intermediate monocytes (β₁ = − 0.12, 95% CI [–0.23 to − 0.01], *p* = 0.033) within the observation period.

Concerning T cells, a non-significant relative decrease in Th2 cells was observed over time (day 1 vs. day 11; 45.5% vs. 40.0%; *p* = 0.075), as well as a relative increase in Th17 cells (day 1 vs. day 11; 36.3% vs. 40.5%; *p* = 0.47). The mixed-effects model revealed this trend to be a significant temporal decrease in Th2 cells (β₁ = − 0.87, 95% CI [–1.61 to − 0.13], *p* = 0.021). For Th17, Th1, and Tregs, no significant longitudinal changes were detected in the mixed-effects model.

### Monocyte and T cell subpopulations in aSAH patients with vs. without angiographic cerebral vasospasm

**Fig. 3:**
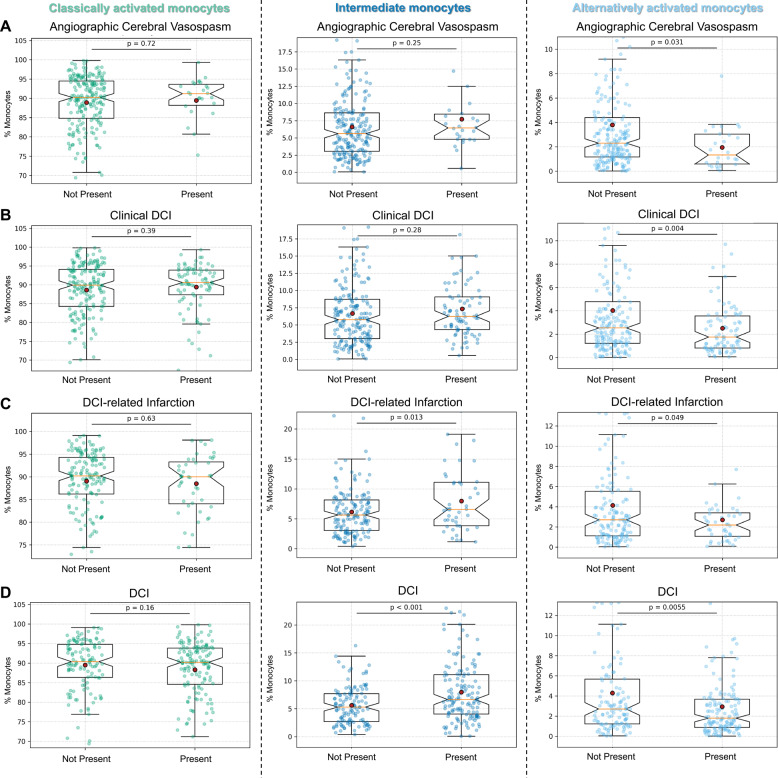
Monocyte subtype distribution in angiographic cerebral vasospasm and DCI Representation of the relative number of alternatively activated monocytes (left column), intermediate monocytes (middle column), and alternatively activated monocytes (right column) for patients with aSAH, categorized by the presence (*n* = 17) or absence (*n* = 58) of angiographic cerebral vasospasm (**A**), presence (*n* = 24) or absence (*n* = 51) of clinical DCI (**B**), presence (*n* = 32) or absence (*n* = 43) cerebral infarction due to DCI (**C**), and presence (*n* = 41) or absence (*n* = 34) of overall DCI (**D**). Data are presented as mean ± SD. Significance was defined as a *p*-value < 0.05 for comparisons between the mean of the subpopulations before the event in affected patients and the mean of the subpopulations over the entire observation period in unaffected patients. The red circle symbolizes the mean, and the orange line indicates the median

In 17 patients, angiographic cerebral vasospasm was observed during the observation period, which was preceded by a shift in monocyte and T cell subpopulations. Patients with angiographic cerebral vasospasm showed a significantly lower relative number of alternatively activated monocytes (3.8% vs. 2.0%; *p* = 0.031, Cohen’s d=−0.57, CI [–0.98 to − 0.15]) (Fig. 3A). This association remained evident in multiple logistic regression although statistical significance was not reached (*p* = 0.081). Regarding T cell subpopulations, a significantly increased relative number of Th1 T cells was observed (Supplementary Fig. 1). The exact numerical values are provided in Table 2.

### Monocyte and T cell subpopulations in aSAH patients with vs. without DCI

Patients who developed DCI (*n* = 41) exhibited a distinct and significant shift in monocyte subpopulations compared to aSAH patients without DCI. Specifically, prior to or at the onset of DCI, these patients showed an increased relative proportion of intermediate monocytes (5.6% vs. 8.0%; *p* < 0.0001; Cohen’s d = 0.53, CI [0.29 to 0.78]) and a decreased relative number of alternatively activated monocytes (4.3% vs. 2.9%; *p* = 0.006; Cohen’s d=−0.34, CI [–0.58 to − 0.10]) (Fig. 3D). This shift remained significant even when considering the individual subcategories of DCI (Table 2; Fig. 3B/C). These associations remained statistically significant in multiple logistic regression models (*p* = 0.007 for clinical DCI, *p* = 0.012 for cerebral infarction due to DCI, *p* = 0.007 for pooled DCI). For the T cell subpopulations, only non-significant trends were observed, as depicted in Supplementary Fig. 1. The exact numerical values are provided in Table 3.

**Table 3:** Relative distribution of T cell subtypes

	Th1	Th2	Th17	Tregs	
	% of T cells (± SD)				n
**Control**	15.08 (8.5)	75.2 (11.6)	6.2 (7.7)	5.9 (2.7)	20
**Time Course**					
Day 1	9.5 (9.0)	45.5 (29.9)	36.3 (28.4)	8.0 (4.4)	72
Day 4	11.5 (10.2)	51.0 (29.7)	29.6 (27.4)	7.0 (3.7)	74
Day 7	9.4 (9.1)	42.7 (32.1)	38.4 (30.7)	7.7 (4.9)	64
Day 11	8.6 (9.7)	40.0 (32.8)	40.5 (30.8)	7.5 (4.2)	60
**Outcome**					
Favorable	9.7 (10.5)	49.1 (30.5)	34.4 (29.8)	6.6 (3.5)	35
Poor	9.5 (7.5)	39.7 (28.5)	39.4 (27.3)	9.5 (4.5)	28
Death	9.1 (5.7)	53.7 (25.2)	30.7 (22.3)	9.3 (5.3)	8
**Angiographic Cerebral Vasospasm**					
Not Present	9.9 (8.4)	45.1 (25.7)	36.1 (24.3)	7.6 (3.6)	58
Present	11.9 (8.1)	47.0 (23.2)	31.4 (20.4)	7.7 (3.5)	17
**Clinical DCI**					
Not Present	10.0 (8.5)	46.1 (25.8)	35.3 (24.3)	7.6 (3.7)	51
Present	11.2 (7.9)	44.2 (23.5)	34.8 (21.9)	7.6 (3.2)	24
**DCI related Infarction**					
Not Present	10.2 (9.0)	47.2 (25.5)	33.9 (23.6)	7.4 (3.5)	43
Present	10.4 (7.4)	43.2 (24.6)	36.9 (23.6)	7.6 (3.2)	32
**DCI**					
Not Present	11.0 (9.8)	48.2 (26.5)	32.3 (24.7)	7.5 (3.7)	34
Present	9.7 (6.8)	43.2 (23.8)	37.6 (22.4)	7.7 (3.5)	41

Representation of the relative proportions of monocyte subgroups among all monocytes in percentage ± *SD* standard deviation. Outcome = mRS 6 months post bleeding (favorable = 0–2; poor = 3–5, death = 6). *DCI* delayed cerebral ischemia, *n* number of patients

### Relationship between monocyte and T cell subpopulations with the outcome in aSAH patients

For the distribution of monocyte subpopulations, no significant correlation was observed with clinical outcomes at 6 months after bleeding (classically activated: *p* = 0.26, intermediate: *p* = 0.43, alternatively activated: *p* = 0.54) (numerical values: Table 2). Similarly, T cell subpopulations showed no significant correlation with the outcome 6 months after bleeding (Th1: *p* = 0.65, Th2: *p* = 0.77, Th17: *p* = 0.83. Tregs: *p* = 0.19). The exact numerical values are provided in Table 3.

## Discussion

It is well-established that inflammation plays a key role after aSAH, but no definitive therapeutic intervention has proven successful in modulating this response. Monocytes and T cells play a critical role in the sterile inflammation seen after aSAH [8, 9, 14, 25–27], with evidence from various diseases, including infections, cardiovascular and inflammatory disorders, cancer, and autoimmune conditions, suggesting that their subpopulations exert distinct, sometimes opposing effects on the inflammatory response [28–31]. These subpopulations of monocytes and T cells secrete, among other factors, different and varying amounts of cytokines, exerting partially opposing effects, whereby inflammation can be either exaggerated or attenuated depending on their polarization to different subgroups [28–31]. To date, however, despite their eminent role in initiating and upholding an inflammatory response, it is not known how monocyte and T cell subpopulations behave after aSAH and their role in post-hemorrhagic complications is not fully understood. By analyzing these immune cell subtypes, our study contributes to filling this knowledge gap, providing new avenues for diagnostic and therapeutic approaches.

In this prospective controlled cohort study, we demonstrate a distinct and early shift in monocyte and T-cell subpopulations toward a pro-inflammatory milieu in patients following aSAH, compared to healthy controls. Specifically, within 24 h post-hemorrhage, we observed a 52% reduction in the relative proportion of anti-inflammatory alternatively activated monocytes, a 39% decrease in anti-inflammatory Th2 cells, and a remarkable 585% increase in the proportion of inflammatory Th17 T cells (Fig. 2A/C). Although there was also a 37% increase in regulatory T cells and a 36% reduction in pro-inflammatory Th1 cells, the overall profile indicated a marked shift toward a pro-inflammatory immune response, not only in relative proportions but also in absolute numbers (Fig. 2A/C). This early and pronounced shift in immune cell subpopulations aligns with prior studies linking post-aSAH inflammation to elevated pro-inflammatory cytokine levels and immune cell activation [8, 9, 14, 25–27]. Our study provides detailed insights into immune cell dynamics, confirming previous findings on Th17 increases in a small cohort of 15 patients [32], while adding granularity by focusing on high-resolution data of systemic cellular response in a larger prospective cohort and combining clinical, radiological, and immunological data. Moreover, the pronounced increase in Th17 cells and decrease in Th2/Treg cells—evident within 24 h post‑aSAH—is reminiscent of autoimmune‑like, IL‑17‑driven mechanisms that, in other CNS diseases, have been linked to blood–brain barrier disruption and immune infiltration [33]. While our study cannot distinguish systemic from central effects, these findings raise the possibility that similar IL‑17‑mediated immune processes may occur intracerebrally after aSAH. However, we acknowledge that this interpretation remains speculative, as we did not collect serum cytokine data (e.g., IL-6 or IL-17 A) to support this hypothesis directly.

Over the course of time following aSAH, we observed trends in the composition and distribution of monocyte and T-cell subpopulations. While initial linear regression suggested non-significant trends, linear mixed-effects modeling revealed a gradual recovery of initially reduced alternatively activated monocytes, while intermediate monocytes declined significantly over time (Fig. 2B). Additionally, a significant longitudinal decline in Th2 cells and a non-significant increase in Th17 cells was observed (Fig. 2D). These shifts suggest that while the initial monocyte-driven inflammatory response may attenuate over time, T-cell subpopulations may continue to sustain a pro-inflammatory environment. Although speculative due to the non-significant trends, these observations are consistent with the expected trajectory of an evolving immune response, where innate immune cells like monocytes dominate early inflammation, followed by sustained T-cell activity as part of the adaptive immune responses.

The most significant finding of this study, however, relates to the correlation between monocyte subpopulation dynamics and the onset of complications. Notably, patients who developed angiographic cerebral vasospasm or delayed cerebral ischemia exhibited a significant reduction in alternatively activated monocytes prior to the onset of these complications (Fig. 3), with meaningful effect sizes. This finding underscores the potential role of monocyte subpopulation polarization in the pathophysiology of aSAH-related complications. The observed shift towards a pro-inflammatory milieu, characterized by a loss of alternatively activated monocytes, represents a novel and pivotal finding. This observation aligns well with previous research identifying specific cytokine patterns [34, 35] and increase in monocytes and T cells [9, 25, 36, 37] as contributors to vasospasm and DCI, but offers new insight by identifying the specific monocyte subpopulations involved. While these results suggest that monitoring monocyte subpopulations might help identify patients at higher risk for complications, the predictive utility of these findings remains to be determined and requires confirmation in future studies designed for this purpose. Importantly, these associations remained statistically significant for cerebral infarction due to DCI, clinical DCI, and pooled DCI even after adjustment for age, sex, WFNS grade, and modified Fisher grade in multiple logistic regression models, further supporting the potential clinical relevance of this immune marker. No significant differences in immune profiles were observed between patients with and without anti-infective or immunomodulatory treatment, suggesting aSAH-specific rather than treatment-driven immune alterations.

Moreover, although peripheral immune cells such as monocytes and T cells are known to cross the blood–brain barrier under pathological conditions, our study did not assess the presence or activation of these cells within the cerebrospinal fluid or brain parenchyma. As such, the findings should not be overinterpreted as direct evidence of neuroinflammation. Instead, they highlight potential peripheral immune correlates of aSAH-related complications. However, the prospective nature of our study, along with the high-quality, detailed immune profiling, adds weight to these findings and highlights the potential relevance of immune monitoring after aSAH. Future investigations incorporating CSF sampling or brain tissue studies will be essential to determine whether the observed peripheral immune changes are mechanistically linked to intracerebral immune mechanisms.

Despite the importance of these findings, we found no significant correlation between immune subpopulations and long-term outcomes at 6 months. This may be due to the standardized care all patients received [38], with timely treatment of complications, potentially minimizing their impact on outcomes. However, it is possible that in cohorts with different treatment protocols or less standardized care, a correlation between immune subpopulations and outcomes may emerge. Additionally, certain differences in outcomes, such as aspects of quality of life or psychological well-being, may exist but were not captured by the modified Rankin Scale used in this study. This scale is relatively coarse, may be subject to individual perception, and does not reflect the complexity of neurocognitive recovery or patient-centered outcomes. Other relevant endpoints such as ICU length of stay, cognitive status, or secondary complications were not systematically assessed and should be addressed in future studies. It must also be considered that the relatively small sample size may have limited the statistical power to detect weaker correlations between immune profiles and long-term functional outcomes. Therefore, early immune signatures may still hold value as intermediate biomarkers for patient monitoring or therapy stratification.

When considering potential therapeutic implications, alternatively activated monocytes may represent a promising avenue for further investigation. Given their significant reduction both shortly after aSAH and before the onset of complications, strategies aimed at preventing the loss of these cells or promoting their polarization could have effects on the inflammatory process and its sequelae [39]. The important role of alternatively activated monocytes has not only been demonstrated in other diseases [14], but animal models have already shown the therapeutic potential of modulating alternatively activated monocytes, such as through mesenchymal stem cell-derived extracellular vesicles, which induced alternatively activated monocyte polarization and exerted neuroprotective effects in experimental aSAH [40, 41]. Our study provides initial human data suggesting that preserving or enhancing the function of these cells might influence the inflammatory cascade after aSAH. However, whether these peripheral changes reflect central immune processes, and whether they can be safely and effectively targeted in patients, and if this results in a reduction of Complications like angiographic cerebral vasospasm and DCI, requires further research in translational models and larger clinical cohorts.

## Limitations

This monocentric study included diverse ethnicities but may not be generalizable to all populations. The single-center design, however, with standard conditions and a standardized treatment protocol reduces heterogeneity and bias. The small sizes of some immune subpopulations and the high interpersonal variability also introduced variability, complicating data pooling and analysis. To address this, a larger patient cohort or a baseline would be necessary. Yet, obtaining a baseline before aSAH is not possible due to its unpredictability prior to the bleeding. Especially when considering the T cell subpopulations, the necessity for larger study populations becomes evident to potentially capture significant changes in these cells, which seem to be present but to a much lesser extent than in the monocyte subpopulations. Selection bias due to the consent process cannot be ruled out, though we believe the study population to be reasonably homogeneous. Furthermore, clinical DCI is a diagnosis of exclusion and inherently prone to confounding. However, through the prospective design and detailed clinical monitoring of all patients, including targeted diagnostics where appropriate, we aimed to minimize misclassification. Nevertheless, residual diagnostic uncertainty cannot be excluded and may have influenced the observed associations and factors such as systemic stress responses and other unmeasured variables may have influenced the observed associations and cannot be fully excluded. No formal correction for multiple comparisons was applied in this exploratory study. Given the relatively small sample size and the high variability inherent to immune cell populations, strict adjustment methods such as Bonferroni correction would have substantially increased the risk of type II errors—i.e., failing to detect true biological associations. Furthermore, given the relatively small sample size in the subgroup analyses, particularly for patients with angiographic vasospasm, the statistical power is limited and the risk of type I error must be acknowledged. Therefore, results should be interpreted cautiously and considered hypothesis-generating pending validation in larger cohorts.

## Conclusion

This prospective cohort study demonstrates a distinct early shift in the peripheral immune profile after aSAH, characterized by a reduction in alternatively activated monocytes and an imbalance in T cell subpopulations suggestive of autoimmune-like responses. The consistent association between reduced alternatively activated monocytes and major complications like cerebral vasospasm and DCI, suggests a promising immunological marker and potential therapeutic target in the pathophysiology of aSAH. Validation in larger cohorts and clarification whether the observed peripheral changes reflect corresponding immune processes within the central nervous system will be key steps toward translating these findings into clinical application.

## Supplementary Information

Supplementary Material 1.

## Data Availability

Any data sharing requires prior review and approval by the local institutional ethics committee. If deemed ethically acceptable, fully anonymized data will be made available by the corresponding author upon reasonable request. Any secondary use beyond the scope of the original study requires separate approval by the corresponding author. Statistical/analytic code and supporting documentation will also be available upon request.

## Abbreviations

ACA: Anterior Cerebral Artery
ACOM: Anterior Communicating Artery
ADP: Adenosine Diphosphate
AICA: Anterior Inferior Cerebellar Artery
ANOVA: Analysis of Variance
aSAH: Aneurysmal Subarachnoid Hemorrhage
BA: Basilar Artery
CT: Computed Tomography
DCI: Delayed Cerebral Ischemia
EBI: Early Brain Injury
EDTA: Ethylenediaminetetraacetic Acid
FACS: Fluorescence-Activated Cell Sorting
FBS: Fetal Bovine Serum
FS: Forward Scatter
ICA: Internal Carotid Artery
MCA: Middle Cerebral Artery
PBS: Phosphate-Buffered Saline
PCA: Posterior Cerebral Artery
PCOM: Posterior Communicating Artery
PFA: Paraformaldehyde
PICA: Posterior Inferior Cerebellar Artery
SAH: Subarachnoid Hemorrhage
SCA: Superior Cerebellar Artery
SD: Standard Deviation
SS: Side Scatter
STROBE: Strengthening the Reporting of Observational Studies in Epidemiology
WFNS: World Federation of Neurosurgical Societies

## References

[1] Nieuwkamp DJ, Setz LE, Algra A, Linn FH, de Rooij NK, Rinkel GJ. Changes in case fatality of aneurysmal subarachnoid haemorrhage over time, according to age, sex, and region: a meta-analysis. Lancet Neurol. 2009;8:635–42. 10.1016/s1474-4422(09)70126-7. 19501022 10.1016/S1474-4422(09)70126-7

[2] van Lieshout JH, Dibue-Adjei M, Cornelius JF, Slotty PJ, Schneider T, Restin T, et al. An introduction to the pathophysiology of aneurysmal subarachnoid hemorrhage. Neurosurg Rev. 2018;41:917–30. 10.1007/s10143-017-0827-y. 28215029 10.1007/s10143-017-0827-y

[3] Osgood ML. Aneurysmal subarachnoid hemorrhage: review of the pathophysiology and management strategies. Curr Neurol Neurosci Rep. 2021;21:1–11. 10.1007/s11910-021-01136-9 34308493

[4] Hofmann BB, Fischer I, Donaldson DM, Abusabha Y, Karadag C, Muhammad S, et al. Evaluation of MTT heterogeneity of perfusion CT imaging in the early brain injury phase: an insight into aSAH pathopysiology. Brain Sci. 2023;13:824. 10.3390/brainsci13050824. 37239296 10.3390/brainsci13050824 PMC10216289

[5] van Gijn J, Kerr RS, Rinkel GJ. Subarachnoid haemorrhage. Lancet. 2007;369:306–18. 10.1016/s0140-6736(07)60153-6. 17258671 10.1016/S0140-6736(07)60153-6

[6] Lucke-Wold BP, Logsdon AF, Manoranjan B, Turner RC, McConnell E, Vates GE, et al. Aneurysmal subarachnoid hemorrhage and neuroinflammation: a comprehensive review. Int J Mol Sci. 2016;17:497. 27049383 10.3390/ijms17040497 PMC4848953

[7] Schneider U, Xu R, Vajkoczy P. Inflammatory events following subarachnoid hemorrhage (SAH). Curr Neuropharmacol. 2018;16:1385–95. 29651951 10.2174/1570159X16666180412110919 PMC6251050

[8] Coulibaly AP, Provencio JJ. Aneurysmal subarachnoid hemorrhage: an overview of inflammation-induced cellular changes. Neurotherapeutics. 2020;17:436–45. 31907877 10.1007/s13311-019-00829-x PMC7283430

[9] Feghali J, Kim J, Gami A, Rapaport S, Caplan JM, McDougall CG, et al. Monocyte-based inflammatory indices predict outcomes following aneurysmal subarachnoid hemorrhage. Neurosurg Rev. 2021;44:3499–507. 10.1007/s10143-021-01525-1. 33839947 10.1007/s10143-021-01525-1

[10] Yun S, Yi HJ, Lee DH, Sung JH. Systemic inflammation response index and systemic immune-inflammation index for predicting the prognosis of patients with aneurysmal subarachnoid hemorrhage. J Stroke Cerebrovasc Dis. 2021;30:105861. 34034125 10.1016/j.jstrokecerebrovasdis.2021.105861

[11] Li K, Khan D, Fischer I, Muhammad S. Systemic c-reactive protein predicts cerebral vasospasm and delayed cerebral ischemia following aneurysmal subarachnoid hemorrhage: a retrospective observational study. World Neurosurg. 2024. 10.1016/j.wneu.2024.08.095. 39182835 10.1016/j.wneu.2024.08.095

[12] Fischer I, Lala R, Donaldson DM, Schieferdecker S, Hofmann BB, Cornelius JF, et al. Prognostic value of platelet levels in patients with aneurysmal subarachnoid hemorrhage. Sci Rep. 2024;14:16743. 10.1038/s41598-024-67322-0. 39033250 10.1038/s41598-024-67322-0 PMC11271284

[13] Li K, Khan D, Fischer I, Hänggi D, Cornelius JF, Muhammad S. CLR (C-reactive protein to lymphocyte ratio) served as a promising predictive biomarker for cerebral vasospasm in aneurysmal subarachnoid hemorrhage (aSAH): a retrospective cohort study. J Clin Med. 2024. 10.3390/jcm13040940. 38398254 10.3390/jcm13040940 PMC10889261

[14] Muhammad S, Hänggi D. Inflammation and anti-inflammatory targets after aneurysmal subarachnoid hemorrhage. Int J Mol Sci. 2021. 10.3390/ijms22147355. 34298971 10.3390/ijms22147355 PMC8304004

[15] Vukmanovic-Stejic M, Vyas B, Gorak-Stolinska P, Noble A, Kemeny DM. Human Tc1 and Tc2/Tc0 CD8 T-cell clones display distinct cell surface and functional phenotypes. Blood. 2000;95:231–40. 10607707

[16] Qu N, Xu M, Mizoguchi I, Furusawa J-i, Kaneko K, Watanabe K, et al. Pivotal roles of T-helper 17-related cytokines, IL-17, IL-22, and IL-23, in inflammatory diseases. Clin Dev Immunol. 2013. 10.1155/2013/968549. 23956763 10.1155/2013/968549 PMC3728507

[17] van der Veeken J, Gonzalez AJ, Cho H, Arvey A, Hemmers S, Leslie CS, et al. Memory of inflammation in regulatory T cells. Cell. 2016;166:977–90. 27499023 10.1016/j.cell.2016.07.006 PMC4996371

[18] Hofmann BB, Donaldson DM, Fischer I, Karadag C, Neyazi M, Piedade GS, et al. Blood pressure affects the early CT perfusion imaging in patients with aSAH reflecting early disturbed autoregulation. Neurocrit Care. 2023. 10.1007/s12028-023-01683-8. 36802010 10.1007/s12028-023-01683-8 PMC10499698

[19] Hofmann BB, Fischer I, Engel A, Jannusch K, Donaldson DM, Karadag C, et al. MTT heterogeneity in perfusion CT imaging as a predictor of outcome after aneurysmal SAH. AJNR Am J Neuroradiol. 2021. 10.3174/ajnr.A7169. 34083263 10.3174/ajnr.A7169 PMC8367610

[20] Hofmann BB, Karadag C, Rubbert C, Schieferdecker S, Neyazi M, Abusabha Y, et al. Novel insights into pathophysiology of delayed cerebral ischemia: effects of current rescue therapy on microvascular perfusion heterogeneity. Biomedicines. 2023. 10.3390/biomedicines11102624. 37892998 10.3390/biomedicines11102624 PMC10603935

[21] Hofmann BB, Fischer I, Neyazi M, Karadag C, Donaldson DM, Abusabha Y, et al. Revisiting the WFNS score: native computed tomography imaging improves identification of patients with false poor grade aneurysmal subarachnoid hemorrhage. Neurosurgery. 2023. 10.1227/neu.0000000000002715. 37823661 10.1227/neu.0000000000002715

[22] Hofmann BB, Donaldson DM, Neyazi M, Abusabha Y, Beseoglu K, Hänggi D, et al. Clinical outcome prediction of early brain injury in aneurysmal subarachnoid hemorrhage: the SHELTER score. Neurocrit Care. 2023. 10.1007/s12028-023-01879-y. 38030877 10.1007/s12028-023-01879-y PMC10959788

[23] Vergouwen MD, Vermeulen M, van Gijn J, Rinkel GJ, Wijdicks EF, Muizelaar JP, Mendelow AD, Juvela S, Yonas H, Terbrugge KG, et al. Definition of delayed cerebral ischemia after aneurysmal subarachnoid hemorrhage as an outcome event in clinical trials and observational studies: proposal of a multidisciplinary research group. Stroke. 2010;41:2391–5. 10.1161/strokeaha.110.589275. 20798370 10.1161/STROKEAHA.110.589275

[24] Kapellos TS, Bonaguro L, Gemünd I, Reusch N, Saglam A, Hinkley ER, et al. Human monocyte subsets and phenotypes in major chronic inflammatory diseases. Front Immunol. 2019. 10.3389/fimmu.2019.02035. 31543877 10.3389/fimmu.2019.02035 PMC6728754

[25] Mohme M, Sauvigny T, Mader MM-D, Schweingruber N, Maire CL, Rünger A, et al. Immune characterization in aneurysmal subarachnoid hemorrhage reveals distinct monocytic activation and chemokine patterns. Transl Stroke Res. 2020;11:1348–61. 31858408 10.1007/s12975-019-00764-1

[26] Zhang Z, Fang Y, Lenahan C, Chen S. The role of immune inflammation in aneurysmal subarachnoid hemorrhage. Exp Neurol. 2021;336:113535. 33249033 10.1016/j.expneurol.2020.113535

[27] Jin J, Duan J, Du L, Xing W, Peng X, Zhao Q. Inflammation and immune cell abnormalities in intracranial aneurysm subarachnoid hemorrhage (SAH): relevant signaling pathways and therapeutic strategies. Front Immunol. 2022;13:1027756. 36505409 10.3389/fimmu.2022.1027756 PMC9727248

[28] Ożańska A, Szymczak D, Rybka J. Pattern of human monocyte subpopulations in health and disease. Scand J Immunol. 2020;92:e12883. 32243617 10.1111/sji.12883

[29] Strauss-Ayali D, Conrad SM, Mosser DM. Monocyte subpopulations and their differentiation patterns during infection. J Leukoc Biol. 2007;82(2):244–52. 17475785 10.1189/jlb.0307191

[30] Hirahara K, Nakayama T. CD4 + t-cell subsets in inflammatory diseases: beyond the T h 1/T h 2 paradigm. Int Immunol. 2016;28:163–71. 10.1093/intimm/dxw006. 26874355 10.1093/intimm/dxw006 PMC4889886

[31] Eagar TN, Miller SD. 16 - Helper T-Cell subsets and control of the inflammatory response. In: Rich RR, Fleisher TA, Shearer WT, Schroeder HW, Frew AJ, Weyand CM, editors. Clinical immunology (Fifth Edition). London: Elsevier; 2019. pp. 235–e245231.

[32] Chaudhry SR, Kahlert UD, Kinfe TM, Endl E, Dolf A, Niemelä M, et al. Differential polarization and activation dynamics of systemic T helper cell subsets after aneurysmal subarachnoid hemorrhage (SAH) and during post-SAH complications. Sci Rep. 2021;11(1):14226. 10.1038/s41598-021-92873-x. 34244562 10.1038/s41598-021-92873-x PMC8270974

[33] Kebir H, Kreymborg K, Ifergan I, Dodelet-Devillers A, Cayrol R, Bernard M, et al. Human TH17 lymphocytes promote blood-brain barrier disruption and central nervous system inflammation. Nat Med. 2007;13:1173–5. 10.1038/nm1651. 17828272 10.1038/nm1651 PMC5114125

[34] Romoli M, Giammello F, Mosconi MG, De Mase A, De Marco G, Digiovanni A, et al. Immunological profile of vasospasm after subarachnoid hemorrhage. Int J Mol Sci. 2023;24:8856. 37240207 10.3390/ijms24108856 PMC10218712

[35] Dumont AS, Dumont RJ, Chow MM, Lin CL, Calisaneller T, Ley KF, Kassell NF, Lee KS. Cerebral vasospasm after subarachnoid hemorrhage: putative role of inflammation. Neurosurgery. 2003;53:123–33. 10.1227/01.neu.0000068863.37133.9e. discussion 133 – 125. 12823881 10.1227/01.neu.0000068863.37133.9e

[36] Chaichana KL, Pradilla G, Huang J, Tamargo RJ. Role of inflammation (leukocyte-endothelial cell interactions) in vasospasm after subarachnoid hemorrhage. World Neurosurg. 2010;73:22–41. 10.1016/j.surneu.2009.05.027. 20452866 10.1016/j.surneu.2009.05.027

[37] Bacigaluppi S, Ivaldi F, Bragazzi NL, Benvenuto F, Gallo F, D’Andrea A, Severi P, Uccelli A, Zona G. An early increase of blood leukocyte subsets in aneurysmal subarachnoid hemorrhage is predictive of vasospasm. Front Neurol. 2020;11. 10.3389/fneur.2020.587039. 10.3389/fneur.2020.587039 PMC7779675 33408685

[38] Hoh BL, Ko NU, Amin-Hanjani S, Chou SH-Y, Cruz-Flores S, Dangayach NS, Derdeyn CP, Du R, Hänggi D, Hetts SW, et al. 2023 guideline for the management of patients with aneurysmal subarachnoid hemorrhage: A guideline from the American heart association/American stroke association. Stroke. 2023;54:e314–70. 10.1161/STR.0000000000000436. 37212182 10.1161/STR.0000000000000436

[39] Muhammad S, Chaudhry SR, Dobreva G, Lawton MT, Niemelä M, Hänggi D. Vascular macrophages as therapeutic targets to treat intracranial aneurysms. Front Immunol. 2021. 10.3389/fimmu.2021.630381. 33763073 10.3389/fimmu.2021.630381 PMC7982735

[40] Han M, Cao Y, Guo X, Chu X, Li T, Xue H, et al. Mesenchymal stem cell-derived extracellular vesicles promote microglial M2 polarization after subarachnoid hemorrhage in rats and involve the AMPK/NF-κB signaling pathway. Biomed Pharmacother. 2021;133:111048. 33378955 10.1016/j.biopha.2020.111048

[41] Park J, Chang JY, Kim JY, Lee JE. Monocyte transmodulation: the next novel therapeutic approach in overcoming ischemic stroke? Front Neurol. 2020. 10.3389/fneur.2020.578003. 33193029 10.3389/fneur.2020.578003 PMC7642685

