# Supplementary material for: Deep immunophenotyping in aneurysmal subarachnoid hemorrhage: a prospective and controlled clinical study
Source: J Neuroinflammation. 2025 Nov 29;23:33. doi: 10.1186/s12974-025-03578-1 (PMC12837136; doi:10.1186/s12974-025-03578-1)
Supplement: Supplementary file 1 — Supplementary Material 1. [file 12974_2025_3578_MOESM1_ESM.pdf]

Deep Immunophenotyping in Aneurysmal Subarachnoid Hemorrhage: A prospective and controlled clinical study

Björn B. Hofmann, MD, PhD[1*], Dilaware Khan, PhD[1*], Igor Fischer, PhD[1], Daniel Hänggi MD, PhD[2], Sajjad Muhammad MD, PhD[1,3]

[1] Department of Neurosurgery, Medical Faculty and University Hospital Düsseldorf, Heinrich-Heine-University Düsseldorf, Düsseldorf, Germany

[2] Department of Neurosurgery, International Neuroscience Institute, Hannover, Germany

[3] Department of Neurosurgery, University of Helsinki and Helsinki University Hospital, Helsinki, Finnland

* Contributed equally

Corresponding author:

Sajjad Muhammad MD, PhD and Björn B. Hofmann MD, PhD

Department of Neurosurgery, Medical Faculty and University Hospital Düsseldorf, Heinrich-Heine-University Düsseldorf, Moorenstraße 5, Düsseldorf, Germany

Phone: 0049 211 81 07823

e-mail: sajjad.muhammad@med.uni-duesseldorf.de

e-mail: bjoern.hofmann@med.uni-duesseldorf.de

Supplementary Material

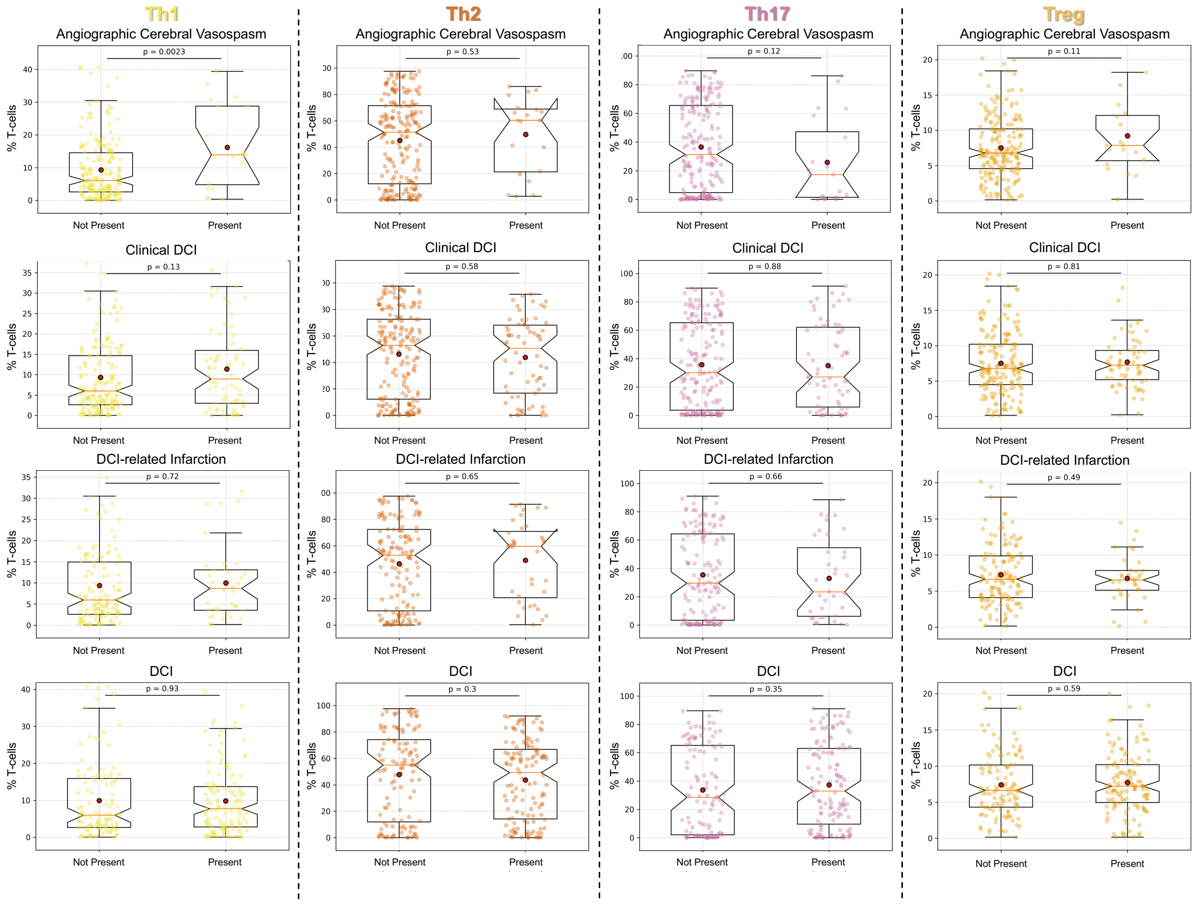

Supplementary Figure 1: T cell subtype distribution in macrovasospasm and DCI

Representation of the Th1 T cells (first column), Th2 T cells (second column), Th17 T cells (third column), and Treg T cells (fourth column) for patients with aSAH, categorized by the presence (n=17) or absence (n=58) of angiographic cerebral vasospasm (A), presence (n=24) or absence (n=51) of clinical DCI (B), presence (n=32) or absence (n=43) cerebral infarction due to DCI (C), and presence (n=41) or absence (n=34) of overall DCI (D). Data are presented as mean ± SD. Significance was defined as a p-value < 0.05 for comparisons between the mean of the subpopulations before the event in affected patients and the mean of the subpopulations over the entire observation period in unaffected patients. The red circle symbolizes the mean, and the orange line indicates the median.

Name	Cat #	Company
APC-H7 Mouse anti-Human CD45	560178	BD Bioscience
APC Mouse Anti-Human CD16	561304	BD Bioscience
FITC Mouse Anti-Human CD14	555397	BD Bioscience
PE-Cy7 Mouse Anti-Human HLA-DR	560651	BD Bioscience

Supplementary Table 1: Monocyte antibodies

Name	Cat #	Company
APC-H7 Mouse anti-Human CD45	560178	BD Bioscience
PE-CF594 Mouse anti-Human CD4	562281	BD Bioscience
PE-Cy7 Mouse Anti-Human CD3	563423	BD Bioscience
FITC Mouse Anti-Human CD127	560549	BD Bioscience
BV510 Mouse Anti-Human CD25	563352	BD Bioscience
APC Mouse Anti-Human CD183	550967	BD Bioscience
PE anti-human CD186 (CXCR6)	Biozol: BLD-356004	Biolegend, order via Biozol

Supplementary Table 2: T-Cell antibodies
